# Serum LRG1 as a diagnostic marker of necrotizing enterocolitis in preterm infants

**DOI:** 10.3389/fped.2026.1769037

**Published:** 2026-05-04

**Authors:** Jingtao Bian, Wenqiang Sun, Yihui Li, Xue Liu, Xinyun Jin, Minqian Zhou, Hanghang Peng, Huawei Wang, Xueping Zhu

**Affiliations:** 1 Department of Neonatology, Children's Hospital of Soochow University, Suzhou, China; 2 Suzhou Medical College, Soochow University, Suzhou, China

**Keywords:** inflammatory response, leucine-rich α-2 glycoprotein 1, necrotizing enterocolitis, premature infants, serum biomarker

## Abstract

**Objective:**

Necrotizing enterocolitis (NEC) remains a leading cause of morbidity and mortality in preterm infants; however, early diagnosis is limited by the lack of reliable circulating biomarkers. This study aimed to evaluate the diagnostic value of serum leucine-rich *α*-2 glycoprotein 1 (LRG1) in NEC.

**Methods:**

LRG1 was identified through an integrative bioinformatics analysis by intersecting differentially expressed genes from two Gene Expression Omnibus datasets (GSE46619 and GSE64801) with the Secreted Protein Database. A nested case-control study was subsequently conducted in preterm infants (<32 weeks' gestation). Serum LRG1 levels were measured using ELISA, and their associations with clinical and laboratory parameters were analyzed. Machine learning approaches were applied for feature selection, followed by multivariable logistic regression. Diagnostic performance was evaluated using receiver operating characteristic curve analysis.

**Results:**

Serum LRG1 levels were significantly elevated in infants with NEC compared with controls. LRG1 was positively correlated with C-reactive protein and neutrophil count, and negatively correlated with plateletcrit. Higher LRG1 levels were independently associated with NEC. In the predictive model, LRG1 demonstrated moderate diagnostic performance (area under the curve = 0.811).

**Conclusion:**

Serum LRG1 is a candidate adjunctive biomarker associated with NEC in preterm infants. However, these findings remain exploratory and require validation in larger, multicenter studies.

## Introduction

Necrotizing enterocolitis (NEC) remains a major cause of gastrointestinal morbidity and mortality in preterm infants, with an incidence of approximately 5%–12% among very low birth weight infants (1, 2). Despite advances in perinatal and neonatal intensive care that have markedly improved survival, the overall incidence of NEC has not shown a corresponding decline (3). Currently, no specific curative therapy is available, and management remains primarily supportive, including bowel rest, gastrointestinal decompression, antimicrobial therapy, and nutritional support. A considerable proportion of affected infants require surgical intervention, and survivors frequently experience long-term complications such as intestinal adhesions, short bowel syndrome, growth retardation, and neurodevelopmental impairment, collectively imposing a substantial burden on quality of life (4–6). These challenges underscore the critical need for early recognition and reliable biomarkers to enable timely and accurate diagnosis (7, 8).

Leucine-rich *α*-2 glycoprotein 1 (LRG1) is a circulating glycoprotein primarily produced by hepatocytes and neutrophils, and its expression is markedly upregulated in response to inflammatory stimuli (9). Previous studies have shown that serum LRG1 reflects intestinal inflammatory activity in adult inflammatory bowel disease (IBD) and pediatric acute appendicitis and may outperform C-reactive protein (CRP) in certain settings (10, 11). However, its role in NEC remains unknown. Moreover, the development of NEC is typically influenced by multiple factors, and the predictive performance of a single biomarker in clinical diagnosis is often limited. Therefore, integrating novel molecular biomarkers with clinical risk factors and constructing comprehensive predictive models using machine learning approaches has increasingly become an important direction in precision medicine (12–14).

In this study, to identify clinically measurable candidate serum biomarkers, we first performed an integrative bioinformatics analysis of publicly available NEC-related transcriptomic datasets. By intersecting differentially expressed genes (DEGs) with the Secreted Protein Database (SEPDB, https://sysomics.com/SEPDB/), LRG1 was identified as a consistently upregulated secreted protein across multiple NEC-related datasets. Based on its biological relevance and secretory characteristics, we hypothesized that serum LRG1 may serve as a potential biomarker for NEC. Accordingly, we aimed to evaluate the diagnostic value and clinical relevance of serum LRG1 in preterm infants and to explore its potential for early identification of NEC using a robust feature selection strategy that combines LRG1 with relevant clinical parameters.

## Materials and methods

### Study population

This nested case–control study was conducted in premature infants admitted to the Department of Neonatology at the Children's Hospital of Soochow University between 1 September 2023 and 30 June 2025. A total of 153 infants with a gestational age <32 weeks who were admitted within 24 h of birth were considered for inclusion. The study protocol was approved by the Ethics Committee of Children's Hospital of Soochow University (2025CS231), and written informed consent was obtained from all guardians, including permission to use and disclose clinical data, in accordance with the 1964 Helsinki Declaration and its subsequent amendments. The inclusion criteria for NEC cases were as follows: (1) a diagnosis of NEC based on clinical manifestations, laboratory findings, and imaging evidence; and (2) Bell stage II or higher (15). Exclusion criteria included severe congenital malformations, confirmed inherited metabolic diseases or chromosomal abnormalities, refusal of participation by guardians, or incomplete clinical data. Based on these criteria, 40 infants with NEC were enrolled. Forty non-NEC preterm infants hospitalized during the same period were selected as controls using 1:1 individual matching by gestational age (±3 days) and birth weight (±100 g) to improve baseline comparability. Serum samples and clinical data were collected prospectively from all participants.

### Clinical data collection

Clinical data were collected prospectively from medical records and included demographic characteristics, perinatal information, and laboratory parameters. Laboratory parameters were obtained from routine clinical testing and included hematological indices [white blood cell count (WBC), neutrophil count (N), lymphocyte count (LY), monocyte count (MO), hemoglobin (HB), red cell distribution width (RDW), hematocrit (HCT), mean corpuscular volume (MCV), platelet count (PLT), mean platelet volume (MPV), and platelet distribution width (PDW)], inflammatory markers (CRP and plateletcrit), and biochemical indicators [albumin (Alb), total bilirubin (TBil), alkaline phosphatase (ALP), lactate dehydrogenase (LDH), glycocholic acid (GCA), and lactate (LA)]. All laboratory data were obtained from blood samples collected as part of routine clinical care.

### Biological sample collection

Serum samples were prospectively collected from preterm infants at predefined time points, with the first sample obtained within 24 h of birth and subsequent samples collected weekly until death, discharge, or 8 weeks of age. All samples were obtained from residual blood remaining after routine clinical laboratory testing. Blood samples were centrifuged at 3,500 × *g* for 10 min at 4 °C within 2 h of collection, followed by a second centrifugation at 12,000 × *g* for 15 min at 4 °C. The serum supernatant was aliquoted into RNase/DNase-free tubes and stored at −80 °C until further analysis.

### Serum LRG1 assay

For the NEC group, serum samples collected within 3 days prior to the onset of NEC were selected for analysis. NEC onset was defined as the time of the first clinical diagnosis based on the Bell criteria. Control serum samples were matched to those of the NEC group by corrected gestational age (±3 days) to minimize the potential influence of developmental factors. Serum LRG1 concentrations were measured using a commercially available ELISA kit (Elabscience, Wuhan, China) according to the manufacturer's instructions.

### Bioinformatics data analysis

To identify candidate biomarkers detectable in serum and subsequently validated in our clinical cohort, publicly available human intestinal transcriptomic datasets related to NEC were retrieved from the GEO database. Differential expression analysis of microarray data was performed using the “limma” R package, while high-throughput sequencing data were analyzed using the DESeq2 package. Genes with |log2 fold change| > 1 and adjusted *P* < 0.05 were considered DEGs. DEGs were further intersected with SEPDB to identify genes encoding secreted proteins. Gene Ontology (GO) and Kyoto Encyclopedia of Genes and Genomes (KEGG) enrichment analyses were conducted using the clusterProfiler package in R, and the results were visualized using ggplot2.

### Feature selection and machine learning analysis

Candidate variables were derived from the clinical and laboratory parameters collected in the case–control cohort. LASSO regression was performed using the glmnet package (family = “binomial”, alpha = 1), and the optimal penalty parameter was selected by 10-fold cross-validation. Variables with non-zero coefficients at lambda.min were considered candidate features. SVM-RFE was implemented using the e1071 package with a customized procedure, and a linear kernel SVM was applied to identify the optimal feature subset based on the minimum 10-fold cross-validation error. Random forest analysis was conducted using the randomForest package, with variable importance ranked by MeanDecreaseGini. Univariate logistic regression (ULR) was used to estimate odds ratios, 95% confidence intervals, and *P*-values. To improve robustness and reduce model instability in this relatively small dataset, only variables repeatedly selected across multiple feature selection methods were retained for final multivariable modeling. As no independent training/test split or external validation cohort was available, the machine learning analyses were intended for exploratory feature screening rather than for formal predictive model validation.

### Statistical analysis

Continuous variables were tested for normality. Normally distributed data were expressed as mean ± standard deviation (SD) and compared using the independent-samples *t*-test. Non-normally distributed data were presented as median (interquartile range, IQR) and analyzed using the Mann–Whitney *U* test. Categorical variables were compared using the chi-square test or Fisher's exact test, as appropriate. Spearman correlation analysis was used to evaluate associations between serum LRG1 levels and laboratory parameters. Correlations were considered meaningful when |R| > 0.30 and *P* < 0.05. The diagnostic performance of the models was evaluated using receiver operating characteristic (ROC) curve analysis, and the area under the curve (AUC) was calculated using the pROC package in R. Nomogram construction, calibration analysis, decision curve analysis, and clinical impact curve analysis were performed using the rms and rmda-related workflows in R. A two-sided *P*-value <0.05 was considered statistically significant.

## Results

### Identification of LRG1 as a candidate secreted biomarker in NEC-related transcriptomic datasets

**Figure 1:**
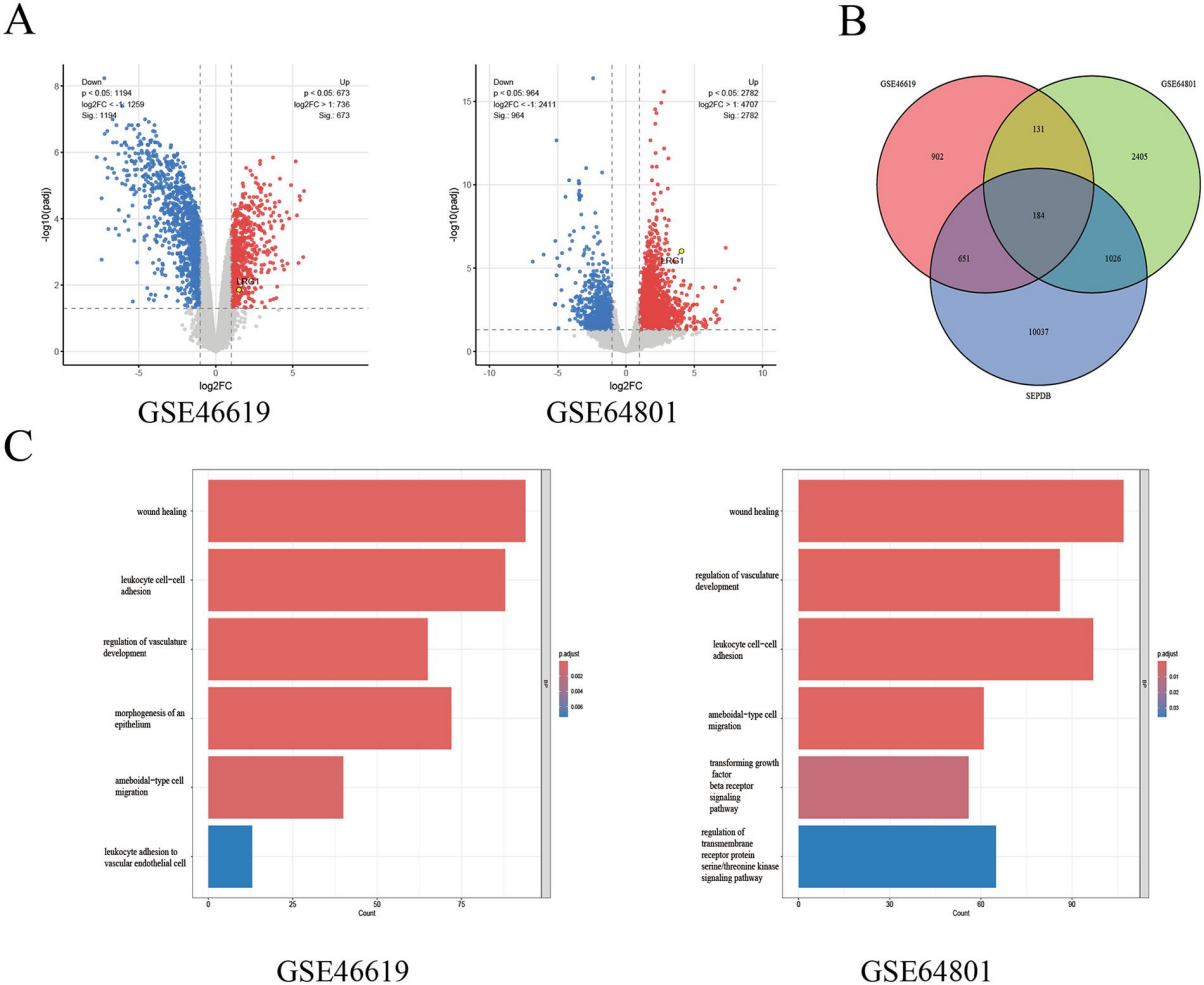
Bioinformatics identification of LRG1 in NEC-related datasets. **(A)** Differentially expressed genes in GSE46619 and GSE64801. **(B)** Overlap of differentially expressed genes with the Secreted Protein Database. **(C)** Functional enrichment analysis of intersecting candidate genes.

Differential expression analysis identified 673 upregulated and 1,194 downregulated genes in GSE46619 and 2,782 upregulated and 964 downregulated genes in GSE64801 (Figure 1A). Intersection with the Secreted Protein Database identified 184 genes encoding secreted proteins (Figure 1B). Among these candidates, LRG1 was prioritized for further validation because it was consistently upregulated in both datasets and encodes a secreted protein, making it a clinically accessible candidate for serum-based detection. GO enrichment analyses further showed that these candidate genes were mainly involved in inflammation-related biological processes, including ameboidal-type cell migration, wound healing, and leukocyte cell–cell adhesion (Figure 1C). In addition, GO analysis of GSE64801 revealed enrichment in the transforming growth factor-beta (TGF-*β*) receptor signaling pathway.

### Clinical baseline characteristics of the NEC and non-NEC groups

Among the NEC group, there were 21 boys and 19 girls, with a median gestational age of 29.6 weeks (28.7–30.2 weeks) and a median birth weight of 1,250 g (998–1,400 g). The median age at NEC onset was 22 days (13–32 days). No significant differences were observed between the NEC and control groups in maternal characteristics, baseline demographics, or comorbidities (*P* > 0.05; Table 1). However, compared with non-NEC controls, infants with NEC had a higher frequency of more than two red blood cell (RBC) transfusions and a greater number of days of antibiotic exposure before NEC, while days of enteral feeding before NEC was significantly shorter (*P* < 0.05; Table 1).

**Table 1:** Baseline maternal and infant characteristics, comorbidities, and treatments before NEC onset.

Characteristics	NEC (*n* = 40)	Non-NEC (*n* = 40)	*P*
Maternal characteristics			
Maternal age, median (IQR)	31 (27.25–32.00)	31 (27.00–34.75)	0.721
ART, *n* (%)	3 (7.5)	4 (10.0)	0.692
HDP, *n* (%)	6 (15.0)	10 (25.0)	0.264
Diabetics, *n* (%)	9 (22.5)	8 (20.0)	0.785
Antepartum placental abruption, *n* (%)	2 (5.0)	3 (7.5)	0.644
ROM, *n* (%)	29 (72.5)	28 (70.0)	0.805
ROM more than 24 h, *n* (%)	9 (23.1)	14 (35.0)	0.243
Chorioamnionitis, *n* (%)	0 (0.0)	1 (2.5)	0.314
Antenatal MgSO_4_, *n* (%)	14 (36.0)	11 (27.5)	0.469
Antenatal antibiotics, *n* (%)	7 (17.5)	12 (30.0)	0.189
Antenatal steroids, *n* (%)	28 (70.0)	35 (87.5)	0.056
Cesarean section delivery, *n* (%)	21 (52.5)	23 (57.5)	0.653
Multiple pregnancies, *n* (%)	7 (17.5)	8 (20.0)	0.775
Infant characteristics			
Female, *n* (%)	19 (47.5)	17 (42.5)	0.653
CGA weeks, median (IQR)	29.57 (28.71, 30.25)	29.57 (29.00, 30.28)	0.484
SGA, *n* (%)	10 (25.0)	7 (17.5)	0.412
BW, median (IQR)	1,250.00 (992.50–1,400.00)	1,185 (1,087.50–1,300.00)	0.386
Underlying diseases and comorbidities prior to NEC			
ASD, *n* (%)	29 (74.4)	27 (67.5)	0.502
VSD, *n* (%)	3 (7.7)	2 (5.0)	0.623
Sepsis before NEC, *n* (%)	8 (20.0)	6 (15.0)	0.556
RDS, *n* (%)	22 (55.0)	28 (70.0)	0.166
PDA, *n* (%)	20 (50.0)	12 (30.0)	0.068
Primary treatments administered before NEC			
TPN before NEC, *n* (%)	32 (80.0)	37 (92.5)	0.105
Caffeine before NEC, *n* (%)	27 (67.5)	28 (70.0)	0.809
PS, *n* (%)	18 (45.0)	21 (52.5)	0.502
Intravenous fluid therapy before NEC, *n* (%)	13 (32.5)	18 (45.0)	0.251
Inotropes before NEC, *n* (%)	20 (50.0)	16 (40.0)	0.369
Steroids before NEC, *n* (%)	5 (12.5)	6 (15.0)	0.745
RBC transfusion before NEC, *n* (%)	22 (55.0)	21 (52.5)	0.823
Number of RBC transfusions greater than 2, *n* (%)	15 (37.5)	6 (15.0)	0.022
Probiotics, *n* (%)	17 (42.5)	18 (45.0)	0.822
Antibiotics before NEC, *n* (%)	39 (97.5)	36 (90.0)	0.166
Days of antibiotics before NEC, median (IQR)	15.50 (8.00–29.00)	10.50 (5.50–17.75)	0.011
UVC, *n* (%)	15 (37.5)	17 (42.5)	0.648
PICC before NEC, *n* (%)	27 (67.5)	33 (82.5)	0.121
Ibuprofen for PDA before NEC, *n* (%)	2 (5.0)	5 (12.5)	0.235
Feeding status			
Days of enteral feeding before NEC, days (IQR)	11.5 (0–19.8)	26.5 (0.3–32)	<0.001
Days of fasting before NEC, days (IQR)	2 (0.8–6.2)	1.5 (0–4.0)	0.421

NEC, necrotizing enterocolitis; ART, assisted reproductive technology; HDP, hypertension disorders of pregnancy; ROM, rupture of membranes; SGA, small for gestational age; CGA, corrected gestational age; BW, birth weight; ASD, atrial septal defect; VSD, ventricular septal defect; RDS, respiratory distress syndrome; PDA, patent ductus arteriosus; TPN, total parenteral nutrition; PS, pulmonary surfactant; UVC, umbilical venous catheter; PICC, peripherally inserted central catheter.

### Laboratory findings in NEC and non-NEC groups

As shown in Table 2, no significant differences were observed between the NEC and control groups in most hematological parameters (*P* > 0.05). In contrast, infants with NEC exhibited significantly higher levels of serum RDW, plateletcrit, and CRP than controls (*P* < 0.05).

**Table 2:** Laboratory parameters in preterm infants.

Serum biochemical markers	NEC (*n* = 40)	Non-NEC (*n* = 40)	*P*
LRG1 (ng/mL)	24.28 (18.37–28.92)	15.23 (10.46–18.77)	<0.001
WBC (×10^9^/L)	9.84 (6. 11–11.81)	9.59 (7.39–11.80)	0.456
N (×10^9^/L)	4.26 (1.68–24.66)	3.95 (2.57–23.92)	0.862
LY (×10^9^/L)	4.26 (3.03–17.42)	4.57 (3.36–29.05)	0.473
MO (×10^9^/L)	1.94 (0.91–3.81)	2.18 (1.39–10.05)	0.157
HB (g/L)	111.50 (95.25–131.25)	118.00 (101.25–134.75)	0.256
RDW (%)	16.00 (15.45–18.65)	15.70 (14.50–17.05)	0.021
HCT (%)	0.33 (0.27–0.39)	0.34 (0.30–0.39)	0.436
MCV (fL)	97.00 (93.00–107.00)	102.00 (96.50–108.25)	0.268
PLT (×10^6^/L)	271.50 (167.75–321.50)	263.00 (212.25–319.50)	0.201
MPV (fL)	11.20 (10.40–11.95)	11.20 (10.30–12. 10)	0.911
PDW (%)	14.30 (11.97–16.42)	13.20 (11.60–16.22)	0.475
Plateletcrit (%)	0.26 (0.20–0.39)	0.35 (0.28–0.44)	0.007
CRP (mg/L)	10.26 (7.54–14.00)	5.87 (3.27–8.35)	0.001
Alb (g/L)	30.10 (28.40–32.40)	31.05 (28.47–36.42)	0.199
TBil (μmol/L)	66.15 (43.75–119.42)	111.30 (42.60–144.20)	0.129
ALP (U/L)	248.00 (204.00–340.00)	246.55 (201.25–349.67)	0.761
LDH (U/L)	416.60 (271.30–547.90)	337.65 (275.35–413.90)	0.516
GCA (μg/mL)	5.39 (2.33–14.45)	4.65 (1.26–12.03)	0.356

NEC, necrotizing enterocolitis; WBC, white blood cell; N, neutrophil; LY, lymphocyte; MO, monocyte; HB, hemoglobin; RDW, red cell distribution width; HCT, hematocrit; MCV, mean corpuscular volume; PLT, platelet; MPV, mean platelet volume; PDW, platelet distribution width; CRP, C-reactive protein; Alb, albumin; TBil, total bilirubin; ALP, alkaline phosphatase; LDH, lactate dehydrogenase; GCA, glycocholic acid.

### Serum LRG1 levels and correlations with clinical data

**Figure 2:**
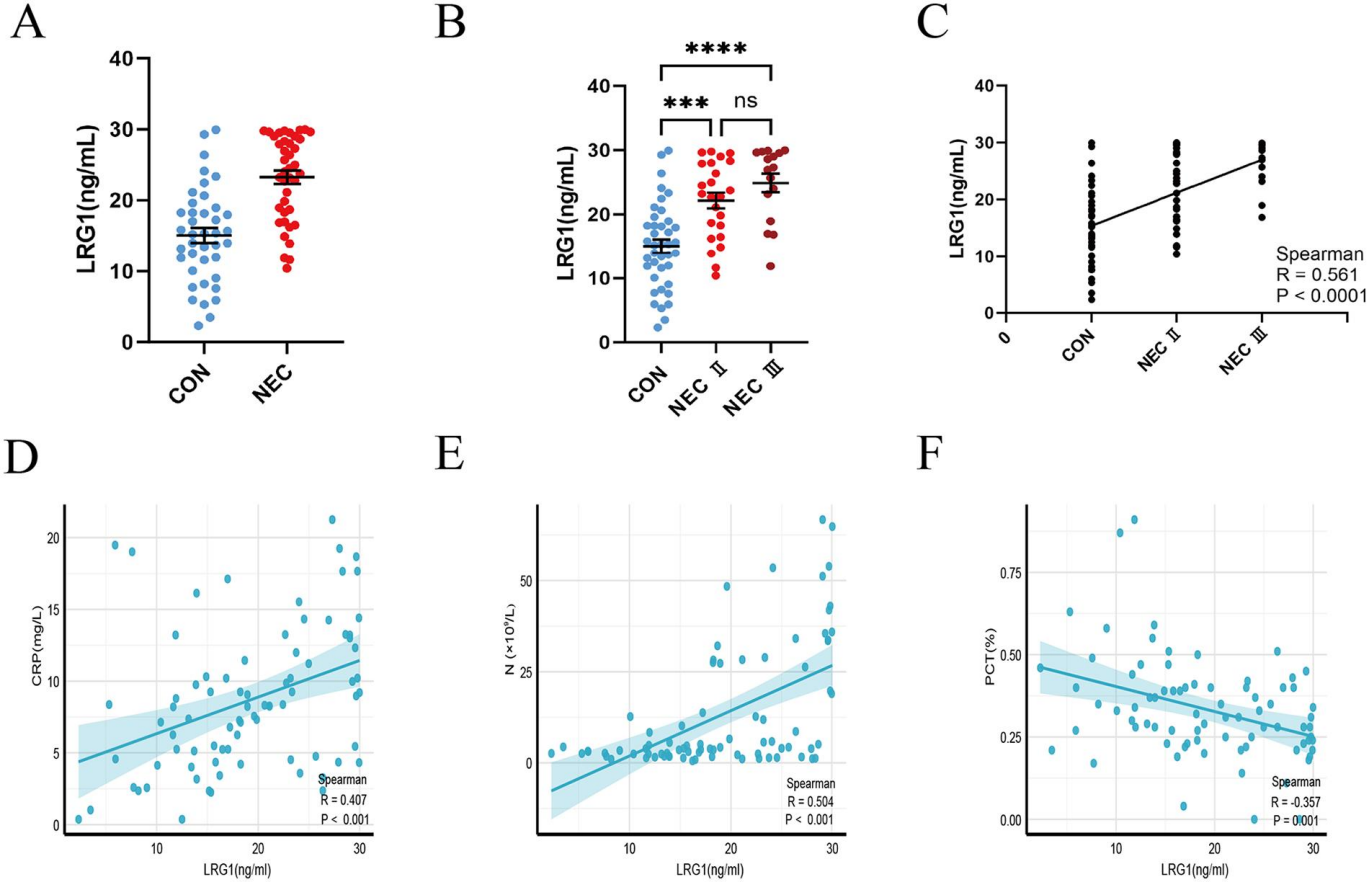
Serum LRG1 expression and clinical correlations. **(A)** Serum LRG1 levels in the NEC and control groups. **(B)** Serum LRG1 levels in NEC subgroups. **(C)** Correlation between serum LRG1 and NEC severity. **(D–F)** Correlations of serum LRG1 with C-reactive protein, neutrophil count, and plateletcrit.

Serum LRG1 levels were significantly higher in the NEC group than in the control group (Figure 2A). However, no statistically significant differences in serum LRG1 levels were observed between stage II and stage III NEC (*P* > 0.05; Figure 2B). Notably, Spearman correlation analysis demonstrated that serum LRG1 levels were positively associated with NEC severity in preterm infants (R = 0.553, *P* < 0.05; Figure 2C). Furthermore, serum LRG1 levels were positively correlated with CRP (R = 0.407, *P* < 0.001) and neutrophil count (R = 0.504, *P* < 0.001) and negatively correlated with plateletcrit (R = −0.357, *P* = 0.001) (Figures 2D–F). No significant correlations were observed with other laboratory parameters.

### Variables associated with NEC in preterm infants <32 weeks of gestational age

**Figure 3:**
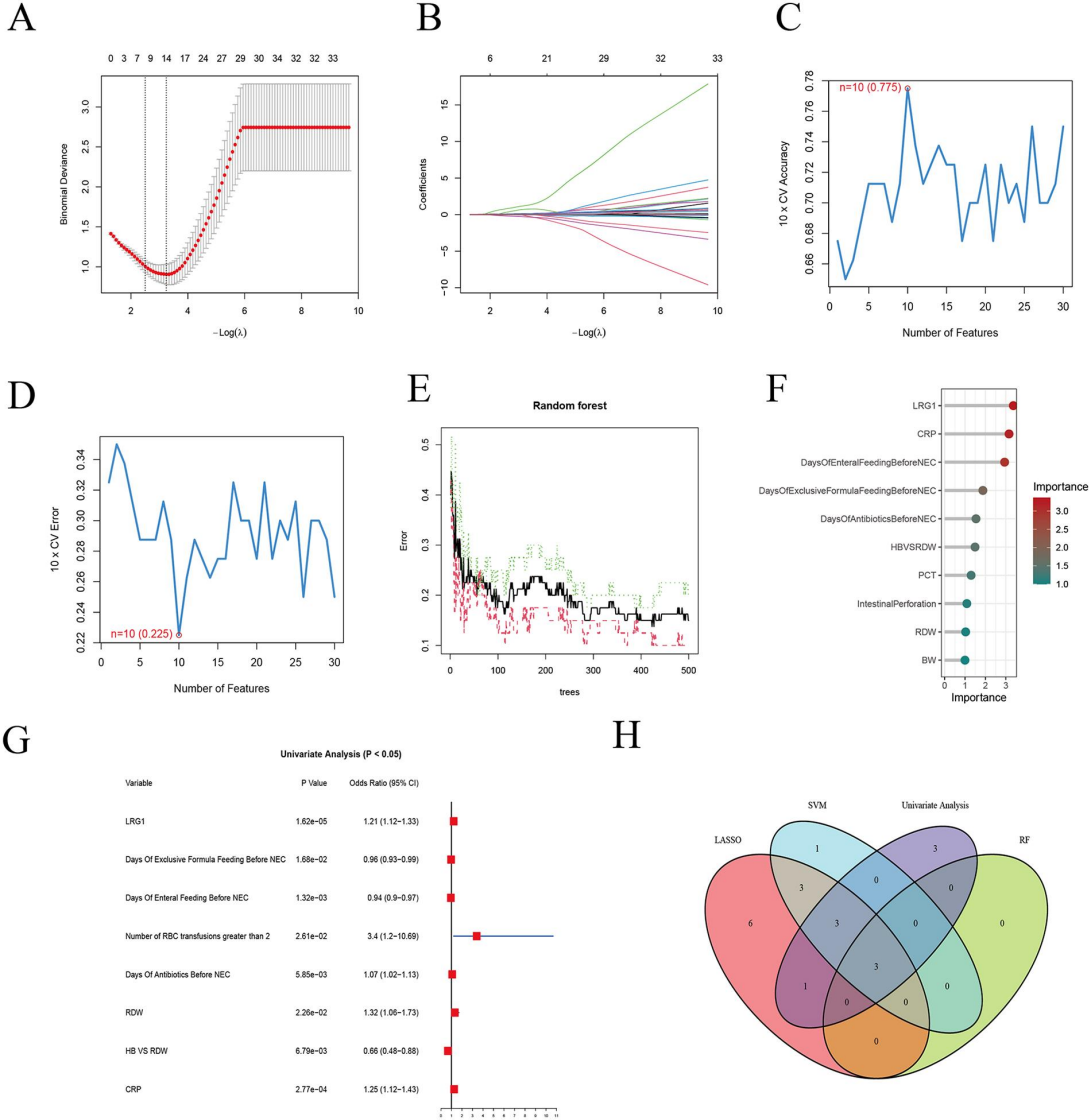
Feature selection for NEC-associated variables. **(A)** Cross-validation for least absolute shrinkage and selection operator regression. **(B)** Coefficient profiles in least absolute shrinkage and selection operator regression. **(C,D)** Support vector machine recursive feature elimination. **(E)** Random forest error rate. **(F)** Random forest variable importance. **(G)** Univariable logistic regression forest plot. **(H)** Overlap of variables identified across feature selection methods.

**Figure 4:**
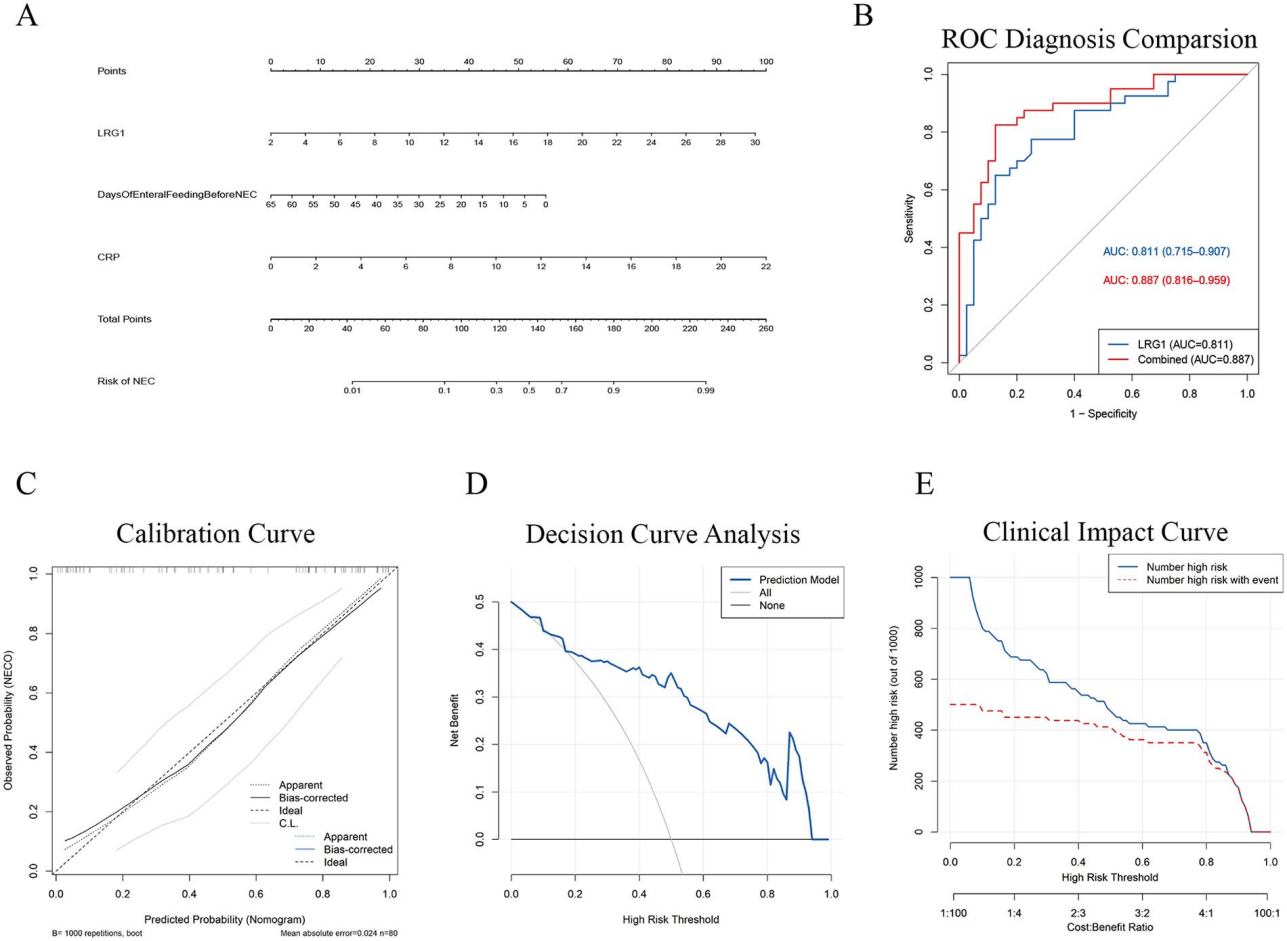
Exploratory combined model for NEC identification. **(A)** Nomogram for estimating the probability of NEC. **(B)** Receiver operating characteristic curves comparing the diagnostic discrimination of serum LRG1 alone and the combined model. **(C)** Calibration curve of the nomogram. **(D)** Decision curve analysis. **(E)** Clinical impact curve.

By integrating the results from multiple models, three common variables were identified: serum LRG1, days of enteral feeding before NEC, and CRP (Figures 3A–H). Multivariate logistic regression analysis demonstrated that days of enteral feeding before NEC was independently associated with lower odds of NEC (OR = 0.934, 95% CI: 0.888–0.983, *P* = 0.009), whereas elevated LRG1 (OR = 1.182, 95% CI: 1.067–1.308, *P* < 0.001) and CRP (OR = 1.242, 95% CI: 1.071–1.442, *P* = 0.004) were independently associated with NEC (Table 3). A nomogram based on the aforementioned variables was constructed (Figure 4A). ROC curve analysis demonstrated that serum LRG1 alone had good discriminatory ability for NEC (AUC = 0.811), while the combined model further improved predictive performance (AUC = 0.887, Figure 4B). Internal validation using bootstrap resampling (B = 1,000), together with calibration, decision curve, and clinical impact curve analyses, demonstrated the robustness and clinical utility of the model (Figures 4C,D).

**Table 3:** Multivariable logistic regression analysis of factors associated with NEC.

Variable	Coefficient	Wald	OR (95%CI)	*P*
LRG1	0.17	10.37	1.182 (1.067–1.308)	<0.001
CRP	0.22	6.86	1.242 (1.071–1.442)	0.004
Days of enteral feeding before NEC	−0.07	8.20	0.934 (0.888–0.983)	0.009

NEC, necrotizing enterocolitis; LRG1, leucine-rich α-2 glycoprotein 1; CRP, C-reactive protein.

## Discussion

NEC remains incompletely understood and is considered multifactorial. Current evidence suggests that intestinal ischemia, microbial dysbiosis, and exaggerated inflammatory responses contribute to mucosal barrier disruption and disease progression (16, 17). Clinically, NEC is primarily diagnosed and staged according to the modified Bell criteria. However, despite its widespread use, this system has several limitations, particularly in the early or atypical stages of the disease, and inconsistencies in case definitions may further contribute to diagnostic variability and research heterogeneity (18, 19). The sensitivity of Bell staging for early detection is suboptimal, which may delay diagnosis and result in missed opportunities for timely intervention, ultimately worsening clinical outcomes (15, 20). In recent years, increasing attention has been directed toward the identification of reliable circulating biomarkers to improve early diagnosis and risk stratification of NEC (7). Against this background, we screened publicly available datasets to identify secreted proteins with potential for serum detection, identified LRG1 as a NEC-related candidate, and demonstrated that serum LRG1 levels were significantly elevated in preterm infants with NEC, showing moderate discriminatory performance. More importantly, the combination of LRG1 with CRP and days of enteral feeding before NEC improved model discrimination compared with LRG1 alone. Taken together, these findings suggest that serum LRG1 may serve as an adjunctive biomarker for the clinical identification of NEC in preterm infants.

LRG1 is a secreted glycoprotein belonging to the leucine-rich repeat family and is primarily synthesized by hepatocytes and neutrophils under physiological and inflammatory conditions (9, 21). Previous studies have shown that circulating LRG1 is associated with inflammatory activity in immune-mediated disorders, including inflammatory bowel disease, in which serum LRG1 correlates with clinical and endoscopic disease activity (10, 22, 23). Moreover, LRG1 has demonstrated diagnostic potential in pediatric inflammatory conditions. Both serum and salivary LRG1 have been reported to differentiate pediatric acute appendicitis from non-specific abdominal pain (24, 25), and cord blood proteomic studies have identified LRG1 as a candidate biomarker for early-onset neonatal sepsis (26). In addition, LRG1 has been shown to be associated with fetal infection and may serve as a potential diagnostic biomarker (27). Collectively, these findings support the biological plausibility of LRG1 as a circulating marker of inflammation and suggest its potential relevance in NEC, a disease characterized by dysregulated immune responses and intestinal injury (16, 17).

LRG1 may be involved in NEC through several potential mechanisms. It has been reported to modulate TGF-β signaling via endoglin-dependent pathways, thereby influencing vascular remodeling and inflammatory responses (28). In addition, its expression can be induced through the IL-6/STAT3 axis, a pathway closely implicated in inflammatory amplification in NEC (29, 30). LRG1 has also been associated with neutrophil activation and recruitment (31). Consistent with these observations, serum LRG1 in our cohort was positively correlated with CRP and neutrophil count and negatively correlated with plateletcrit, suggesting that elevated LRG1 may reflect the systemic inflammatory burden accompanying NEC rather than an isolated biochemical alteration. Nevertheless, these mechanistic links remain inferential in the present clinical study and warrant direct experimental validation.

An important strength of the present work is that it does not evaluate LRG1 in isolation, but rather positions it within the broader context of NEC biomarker research. Although CRP is widely used in neonatal inflammatory conditions and has recognized diagnostic value in NEC, it remains a non-specific acute-phase reactant and may rise in a range of infectious or inflammatory states (32, 33). Similarly, LRG1, a secreted inflammation-related glycoprotein, may provide complementary information beyond conventional inflammatory markers but should not be regarded as a disease-specific marker for NEC, as it may also be elevated in other inflammatory conditions such as neonatal sepsis. Therefore, it should be interpreted in combination with clinical findings and other variables rather than used as a standalone diagnostic marker. Notably, the combined model incorporating LRG1, CRP, and duration of enteral feeding before NEC onset outperformed LRG1 alone, supporting the view that biomarker-based identification of NEC may benefit from integration with clinically relevant variables rather than reliance on a single analyte. In addition, the current study provides a translational evidence chain from bioinformatics-based candidate screening to serum-level clinical validation, which strengthens the rationale for prioritizing LRG1 as a biomarker candidate. The inclusion of days of enteral feeding before NEC in the final model is also biologically plausible, as carefully advanced enteral feeding may promote intestinal maturation, support microbial homeostasis, and mitigate excessive mucosal inflammation (34). Previous studies have likewise suggested that earlier and more gradual enteral feeding strategies may be associated with a lower likelihood of NEC (35, 36). Nevertheless, this variable should be interpreted cautiously, as in a case–control framework, it may also reflect underlying clinical instability before diagnosis, and reverse causality cannot be excluded.

Several limitations should be acknowledged. First, this was a single-center study with a relatively small sample size, which may have limited statistical power, especially for subgroup comparisons. Second, the feature selection and model construction procedures were exploratory, and neither an independent training/test split nor an external validation cohort was available. Therefore, the present findings should not be interpreted as evidence of definitive predictive performance. Third, because this was a nested case–control study and biomarker measurements in the NEC group were obtained shortly before disease onset, the results are more appropriately interpreted as supporting clinical discrimination rather than long-term prospective risk prediction. Fourth, the observed association between serum LRG1 and NEC may also have been influenced by other clinical factors. For example, infants with more severe illness, greater exposure to medical treatments, or early clinical instability before NEC diagnosis may already have had heightened inflammatory responses, which could have affected serum LRG1 levels independently of NEC itself. Finally, broader inflammatory profiling, tissue-level validation of LRG1 expression, and mechanistic experiments were not performed, limiting biological interpretation. Therefore, larger multicenter studies with longitudinal sampling, external validation, and mechanistic investigation are needed to determine the precise clinical utility of serum LRG1 in NEC.

In conclusion, serum LRG1 was significantly elevated in preterm infants with NEC and showed potential value as an adjunctive biomarker for clinical identification, particularly when interpreted together with CRP and days of enteral feeding before NEC; however, these findings remain exploratory and require confirmation in larger multicenter studies.

## Data Availability

The dataset used in this study is publicly available and can be accessed from the Gene Expression Omnibus (GEO) database (https://www.ncbi.nlm.nih.gov/geo/, GSE46619, GSE64801). The clinical datasets generated during and/or analyzed during the current study are not publicly available due to patient confidentiality considerations but are available from the corresponding author on reasonable request following institutional data sharing policies.
